# Visual Attention and Emotion Analysis Based on Qualitative Assessment and Eyetracking Metrics—The Perception of a Video Game Trailer

**DOI:** 10.3390/s23239573

**Published:** 2023-12-02

**Authors:** Eva Villegas, Elisabet Fonts, Marta Fernández, Sara Fernández-Guinea

**Affiliations:** 1 Centre de la Imatge i la Tecnologia Multimèdia, Universitat Politècnica de Catalunya (UPC), 08222 Terrassa, Spain; eva.villegas.portero@citm.upc.edu (E.V.); elisabet.fonts@citm.upc.edu (E.F.); marta.fernandez.ruiz@citm.upc.edu (M.F.); 2 Faculty of Psychology, Universidad Complutense de Madrid (UCM), 28223 Madrid, Spain

**Keywords:** visual attention, human-centered design, eyetracker analysis, vision-based sensing, user experience, emotions analysis, video game

## Abstract

Video game trailers are very useful tools for attracting potential players. This research focuses on analyzing the emotions that arise while viewing video game trailers and the link between these emotions and storytelling and visual attention. The methodology consisted of a three-step task test with potential users: the first step was to identify the perception of indie games; the second step was to use the eyetracking device (gaze plot, heat map, and fixation points) and link them to fixation points (attention), viewing patterns, and non-visible areas; the third step was to interview users to understand impressions and questionnaires of emotions related to the trailer’s storytelling and expectations. The results show an effective assessment of visual attention together with visualization patterns, non-visible areas that may affect game expectations, fixation points linked to very specific emotions, and perceived narratives based on the gaze plot. The innovation in the mixed methodological approach has made it possible to obtain relevant data regarding the link between the emotions perceived by the user and the areas of attention collected with the device. The proposed methodology enables developers to understand the strengths and weaknesses of the information being conveyed so that they can tailor the trailer to the expectations of potential players.

## 1. Introduction

Technology applied to research is advancing rapidly. A qualitative or quantitative review alone is no longer sufficient to comprehend users’ perceptions of different audiovisual experiences. A non-intrusive evaluation is becoming increasingly necessary to avoid interfering with the actual assessment of these experiences.

Studying interfaces, their narratives, and the emotions they convey is analyzed from a user experience evaluation perspective, where various techniques help understand people’s needs and motivations [1,2,3]. If, in this type of study, an understanding of visual attention is added, it can provide information about the lived experience, perceived emotions, and visual fixation areas. Therefore, it is possible to link the thoughts conveyed by users with the actual vision.

This study provides relevant information on the analysis of visual attention along with emotional parameters in order to give developers tools to focus the creation of the trailer according to the players’ perspective. Our case study focuses on the visualization of a video game trailer [4,5], providing useful information for the study of indie video games [6,7], and it involves designing a mixed methodology based on user experience techniques, eyetracking metrics, and emotion assessment.

Additionally, we provide information about studies conducted on video game trailers, visual attention, eyetracking technology, and emotions. We then proceed to explain the methodology along with the techniques applied. Afterward, we present an interpretation of the results for each research phase. Finally, we offer a discussion and a set of conclusions.

### 1.1. Video Game Trailers

The trailer is an original format of the film industry [4]; it is considered a key element in capturing the audience’s attention, evoking a range of emotions, and promoting the film in question, creating anticipation and generating a desire to watch it [8]. Svelch, like other authors [9,10], defines a trailer as an audiovisual paratext that informs the viewer of the existence of a particular video game [4].

Initially, in the video game industry, promotional efforts for early video games focused on exploring hardware functionality [11]. However, in recent times, the approach has shifted to advertising very much akin to movie trailers. Drawing from its origins in the film industry, a video game trailer comes with inherited cinematic quality. This cinematic aspect, in its linear structure, fits well for representing the fictional world of video games, particularly their narratives. Nevertheless, Svelch observes that its role in the video game industry and culture goes beyond marketing [4], and there are other relationships between the trailer and its main material. He proposes a classification based on its relationship with the ludic and cinematic aspects of video games. Recognizing limitations in how a linear paratext like a trailer can represent a nonlinear video game, Svelch classifies them not based on the formal qualities of the trailer but on their relationship between rules and fiction [12]:

- Hardware interaction-focused trailers are common when new technology emerges, as seen in the trailers for the Guitar Hero franchise;
- Performative trailers are audiovisual pieces showing gameplay from the perspective of a viewer watching players interact with the game or the viewer watching the screen;
- Transmedia extension trailers [13] are centered on linear and cinematic representations of the fictional world of the video game;
- Interactive trailers replicate the video game’s interface, focusing more on the ludic aspects to convey its non-linearity.

Following Svelch’s typology, “Spiritfarer” is a trailer with elements of transmedia extension and performative trailer characteristics. One of the desirable characteristics attributed to a video game trailer is representativeness. Representativeness refers to the accuracy of representing the gaming experience of a text through the use of semiotic audiovisual resources [4].

In previous media, such as cinema, reception studies have been carried out focusing on emotions and contrasted with the use of sensors. Prior studies have examined the emotions that trailers provoke, taking into account the storytelling and relying on sensors. Concerning storytelling, Thomsen and Heiselberg [8] examine emotional responses to the content of a movie trailer based on an exploratory study using skin reactions to measure emotional excitement, as well as self-reports on recall, evaluation, and the desire to watch the film. The results indicate that it is not the overall level of excitement but a specific pattern of excitement that allows for the accumulation of memorable scenes.

**Figure 1:**
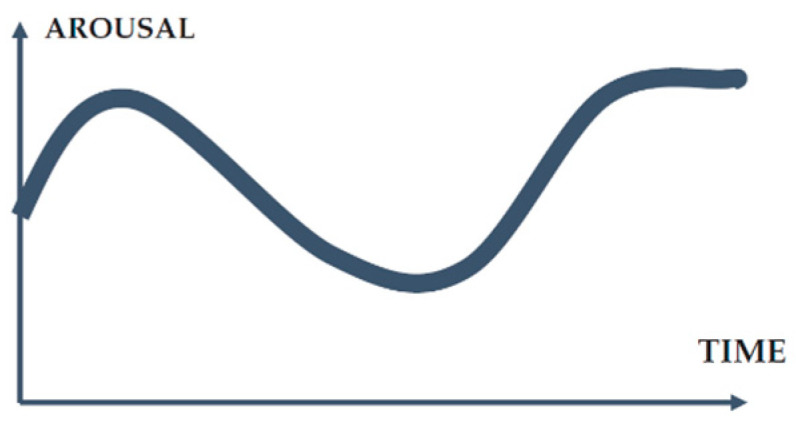
Sketch of the two-peak structure of drama film trailers.

Thomsen and Heiselnerg [8] build on the three-act structure to make a trailer exciting [14]:

- First act: introduces the characters and the movie’s setting;
- Second act: complicates the world of the characters with obstacles to overcome;
- Third act: intensifies conflicts and increases tension, excitement, and humor;
- This structure can be represented as a trajectory that is low at the beginning and gradually ascends toward the end of the trailer. However, to capture the viewer’s attention, Thomsen and Heiselnerg [8] argue that the first part of the trailer should include a peak of excitement for stimuli to gain priority processing. These assumptions can be translated into the structure of two peaks indicated in Figure 1.

Figure 1 shows the level of arousal, corresponding to the degree of brain activation or level of attention, regarding the development time. Emotional arousal or activation is an essential aspect of media entertainment, whether in the form of movies, novels, television programs, music videos, video games, or trailers [15]. Thomsen and Heiselnerg [8] explore the role of arousal when the audience watches dramatic film trailers. They primarily focus on the effect of emotions on the desire to watch the movie. They examine the isolated trailer of the film itself. To conduct this, they adopted a mixed method that combined questionnaires with the galvanic skin response (GSR).

Based on developments in advertising in view of the prediction of the potential impact on the consumer, Christoforou [16] proposed a metric that can be calculated for any narrative video stimulus and can be used in various contexts, including forecasting audience preferences for movies, quantitative evaluation of entertainment pieces, prediction of the impact of movie trailers, and more.

While studies focused on inducing emotions are not aligned with this research, it is worth mentioning that within the study of trailers, it has been studied how they are perceived once the audience is induced into different moods (sad or happy) [17]. Participants in Delvin et al.’s study were induced into positive or negative feelings through watching online content and were then exposed to comedy or serious trailers. They evaluated items such as the intention to watch the movie and attitude toward the movie preview. The results suggest that there is an interaction between mood, trailer type, and genre. Men reported greater purchase intentions with trailers whose genre (serious or comedy) differed from their mood (positive or negative), while women showed a pattern of congruence; that is, women with negative moods responded better to serious trailers.

### 1.2. Visual Attention

Visual attention [18] is defined as a central mechanism for controlling information processing, acting according to the organism’s goals via activating and inhibiting processes, and can be directed toward the senses, memory knowledge structures, and response systems. Visual attention is the result of different information processing activities aimed at detecting and selecting, orienting, and staying vigilant. Visual attention results from three processes: orientation, alertness, and selection. The orientation mechanism allows aligning or focusing attention on different sources of stimulation. Some sources are internal, such as the contents of memory, so that we can direct attention to a memorized visual image. The alertness mechanism enables the organism to reach and maintain a special predisposition to receive a certain type of stimuli. When there is a predisposition to receive a certain type of stimulus, it is detected more quickly than when there is no predisposition. The stimulus will receive a quicker response when it is receiving attention than when it is not. Finally, the stimulus selection mechanism [19] develops over the ongoing activity. For example, when multiple tasks are carried out in parallel, each involves a selection among competing stimuli. The attentional network is involved in numerous activities that allow parallel execution of more than one task, properly distributing cognitive resources to simultaneously attend to more than one set of stimuli that may be related to each of the various tasks executed in parallel [20].

Attention can be voluntary or involuntary, meaning that alertness mechanisms can be activated because a person wants or does not want to pay attention. Some characteristics of external stimuli that capture involuntary attention are power or intensity, movement, visual appearance changes, contrast, the unexpected, component prioritization, or repetition. Attention also takes into account the degree of consciousness required for different types of tasks, determining the use of attentional resources depending on the degree of awareness and, therefore, automation of actions.

Related to attention, there is the orienting reflex [21], which is the immediate response of an organism to a change in its environment, allowing the involuntary attention of individuals to be captured via the unexpected. It is an innate, automatic, and involuntary mechanism. It is activated when a novel stimulus is captured via one of our senses before the signal is cognitively processed or conscious. This reflex causes attentional reactions such as:

- Orientation: movement of the eyes, head, and neck toward where the new object is located;
- Alertness: vigilance;
- Filtering: selection of the stimuli to which attention is paid;
- Concentration: Sustained attention that can be maintained voluntarily.

In terms of cognitive processing, after the automatic reflex, the signal is processed and interpreted to identify the stimulus and decide if it is a stimulus capable of affecting it and how it affects it. If the impact is positive, there is an approach reaction; if it is negative, there is a withdrawal reaction. Concentration refers to a psychological state that involves alertness, orientation, and selection. Alertness involves receiving a certain type of stimuli and not others. Orientation is carried out toward relevant stimuli, while inhibition of irrelevant stimuli occurs simultaneously. The more irrelevant information is inhibited, the greater the concentration. It also involves maintaining this state for an extended period of time.

Having knowledge of what attention is and how it works in humans is useful for designers because it is possible to facilitate people’s focus on the intended content and convey necessary information that is not directly related to the main task, avoiding distraction. Whether a user directs their attention to one component or another is determined through the interaction of processes where attention is intentionally and voluntarily directed and processes where attention is directed according to the stimulus. It is important to note that attention is not solely focused on the visual channel; the auditory channel also influences attention processes.

### 1.3. Eyetracking Technology

The concept of eyetracking refers to a set of technologies that enable the monitoring and recording of how a person gazes at a particular scene or image, specifically, where their attention is fixed, for how long, and the order in which they visually explore it. In this research, the Tobii Pro Lab software, version v 1.217.49450, and Tobii Eye Tracker hardware are used.

**Figure 2:**
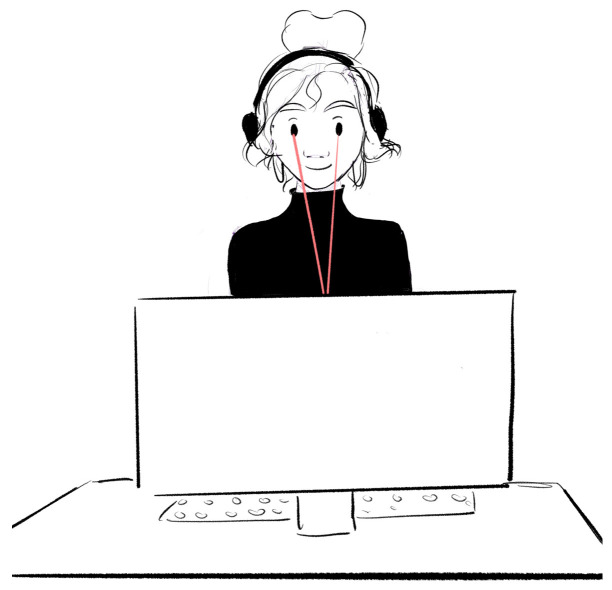
Use of the eyetracking device.

**Figure 3:**
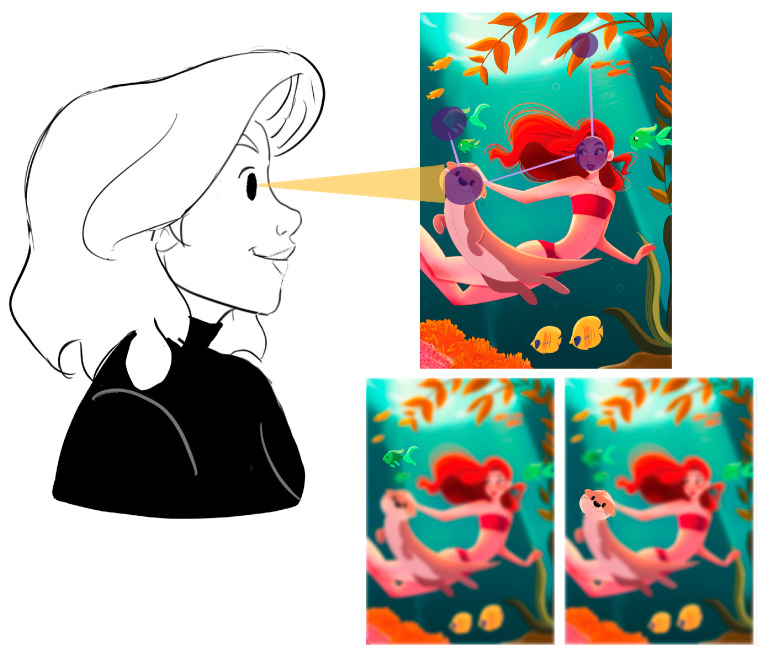
Scene perception. We fixate on the features that attract our visual attention and construct the scene using the information gathered during fixations [23].

The mentioned technology allows for monitoring and recording fixation points of the gaze, the areas viewed, and the order of the visual exploration path. Human vision, illustrated in Figure 2 encompasses a visual field of 220 degrees, divided into three visual areas: the foveal area (1–2 degrees), parafoveal area (2–5 degrees), and peripheral area (6–220 degrees). The area with the highest resolution and visual sharpness is the foveal area, which is captured via eyetracking, registering 50% of the visual information that reaches the brain through the optic nerve [22]. To control visual elements, the eyes perform various eye movements with three functions: placing the information of interest on the fovea, keeping the image fixed on the retina with pursuit movements, and preventing the percentage decay of fixed objects through microsaccades. According to Tobii Technology AB (2010), the eye creates a path of fixations on the most visible elements, which are those that attract the most visual attention. As shown in Figure 3, only when fixations have high levels of attention can the brain combine the details to complete a general view of the scene.

**Figure 4:**
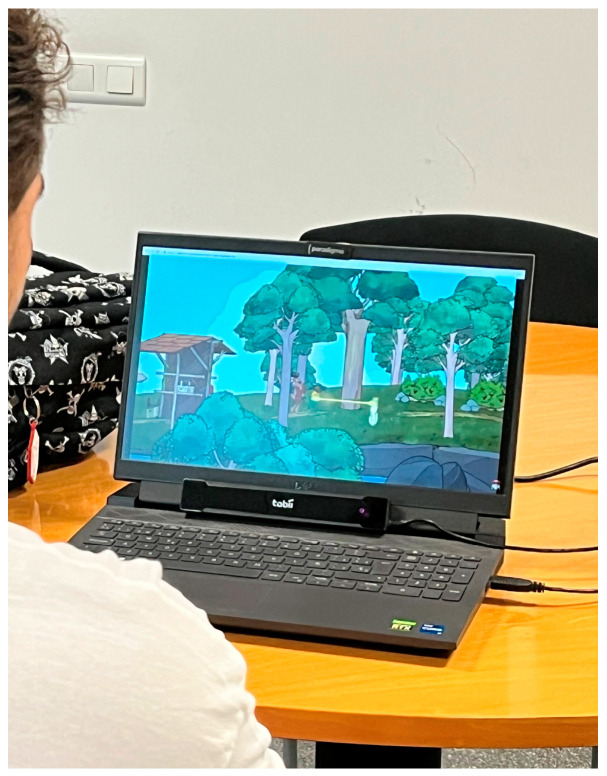
Test room. Participant viewing the trailer with the eyetracking device.

As shown in Figure 4, the eyetracker device used in this study is based on a non-intrusive device connected to the computer, which collects and displays camera data. The information is captured using infrared light, which creates reflections from the cornea and pupil and includes sensors to capture reflection patterns.

Eyetracking technology has been widely used in the fields of human–computer interaction and advertising. It has proven to be a useful tool for quantifying audience preferences in product packaging tests [24], print advertising tests [25], usability studies [26], as well as the visibility of content on web pages [27].

### 1.4. Emotional Behavior

As Robert Plutchik (1980) asserts, an emotion is not simply an emotional state but a complex chain of connected events. This chain begins with a stimulus that encompasses feelings, physiological changes, impulses, and behaviors [28].

This research is based on the emotional behavior [29,30] triggered in users during the viewing of a video game trailer [31,32]. The factors to be considered in this behavior are related to the emotions generated during the trailer’s viewing, the user’s mood, and the pre-associated feelings of the user towards the trailer or the video game [23]. When referring to pre-associated feelings, we consider intrinsic factors, such as motivation, which can be conditioned through extrinsic factors like social and cultural aspects, the product itself (e.g., prior knowledge of previous games from the same author or studio), and the context in which it is used,

The field of emotions is quite extensive, and, according to the authors, it encompasses various types of feelings. This research is based on Robert Plutchik’s wheel of basic emotions [33,34], widely used in psychological studies and affective computing.

**Figure 5:**
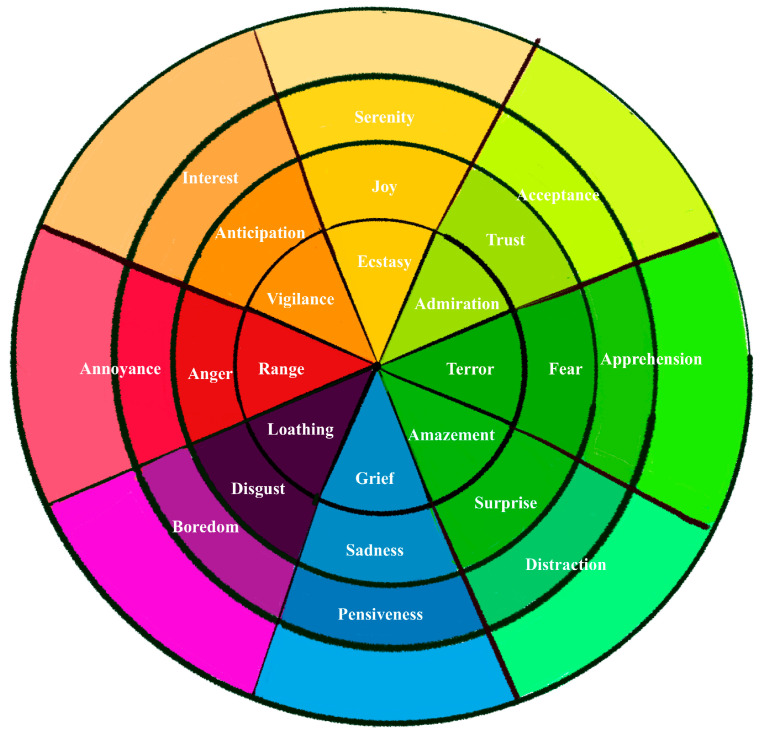
Diagram of Robert Plutchik’s wheel of emotions [35].

Figure 5 shows the wheel of emotions that indicates the eight emotions used for this study:

- Joy: an affective feeling of brief duration that causes a pleasant sensation. It manifests as optimism, triumph, and increased energy. Depending on its degree of intensity, it can vary between serenity, joy, and ecstasy.
- Confidence: a feeling of learning to live with one’s own and other people’s mistakes, prioritizing the positive aspects over the painful ones. Depending on its degree of intensity, it can oscillate between acceptance, trust, and admiration.
- Fear: a feeling of uneasiness caused by real or imagined danger. Depending on its degree of intensity, it may involve apprehension, fear, terror, or panic.
- Surprise: Transitory emotional disturbance caused by an unforeseen, unexpected event. According to its degree of intensity, it can vary between distraction, surprise, and astonishment.
- Sadness: a state of affliction in which the person feels dejection, normally produced due to some setback. Depending on its degree of intensity, it can oscillate between isolation, sadness, grief, or depression.
- Rejection: the repugnance produced by something that is unpleasant and aversive. Depending on its degree of intensity, it may involve boredom, rejection, abhorrence, or disgust.
- Anger: a feeling of annoyance. Depending on its degree of intensity, it can be annoyance, anger, or rage.
- Anticipation: a search for answers, resources, or alternatives to confront conflicts.

At this point, it should be noted that the aim of this research is not to trigger or induce emotions as is done via mood management theory [17], which suggests that an individual will select products or experiences for entertainment to regulate their moods, especially when moods are affected via external stimuli [36,37]. In the context of this study, which is focused on a video game trailer showing concept art, narrative, and game mechanics, visual attention is used as a central mechanism to control information processing through orientation, alertness, and concentration. The eyetracking device, together with the study of the user experience (or, more specifically, the viewing experience of the trailer), allows the creation of a mixed methodology of great value.

## 2. Materials and Method

The study of emotions that arise from the viewing of a video game trailer, as well as the link between these emotions [38], storytelling, visual attention, and the intention to play the video game from which the trailer originates, requires the application of a mixed methodological approach [39].

The evaluated and selected methodological techniques aimed to delve into the participants’ profiles, making them focused and necessary users for the study [40,41,42,43,44]. In this study, there was a divergence while viewing a video game trailer and convergence during the analysis of specific themes [41].

**Figure 6:**
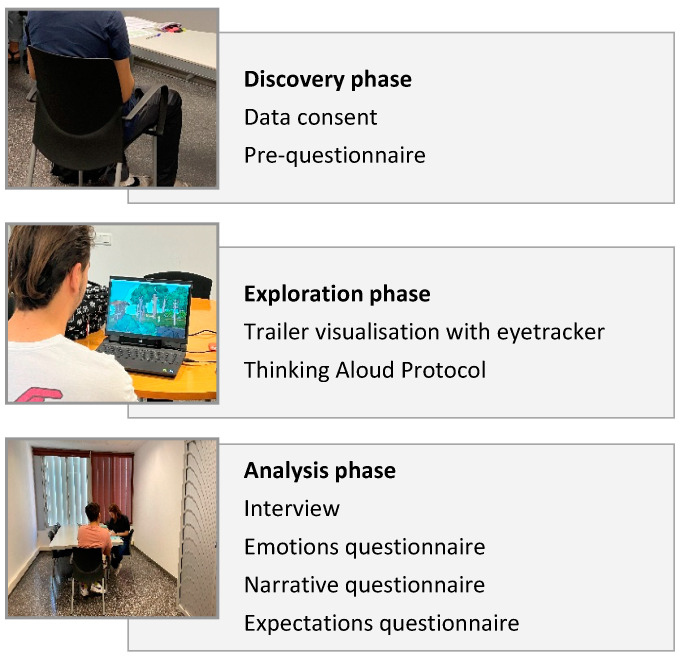
Outline of the methodology used in this research.

As shown in Figure 6, various techniques are applied at each step of the process:

- Step 1: Discovery phase;
- Step 2: Exploration phase;
- Step 3: Analysis phase.

The trailer was intentionally selected for its high emotional and narrative content. The different techniques and phases of this study are described in detail below:

### 2.1. Discovery Phase

After the signing of the data consent document, the objective of the discovery phase was to thoroughly analyze the user’s profile, taking into account factors such as age, knowledge about the components of a game (videogame literacy), factors influencing game choices, aspects valued in a trailer, and knowledge about indie games.

### 2.2. Exploration Phase

During the exploration phase, participants viewed the video game trailer using the eyetracker device, and while doing so, they applied the think aloud protocol by verbally expressing their thoughts. The eyetracker [23] allowed for collecting data related to areas of interest and visual paths within an interface. The device used infrared light to generate patterns of light reflected on the users’ corneas. These patterns of reflected light, along with other visual information, were captured with image sensors, enabling the precise tracking of gaze points on the screen (specifically, where attention is focused, for how long, and the order of their visual exploration).

### 2.3. Analysis Phase

In this phase, the goal was to converge all the information worked on in the previous phases. Once the trailer viewing was completed, the transition was made from divergence to convergence, at which point several techniques were applied.

On the one hand, a closed interview [23,45] was conducted, where the participant described the perceived narrative content of the trailer and their expectations regarding the video game that the trailer promotes. If necessary, the participant could re-watch the video during the interview. Once the interview was completed, the participant was given an emotional assessment questionnaire, created based on Robert Plutchik’s eight emotions [34,46], using a 5-point Likert scale, where 1 represents the lowest rating, and 5 represents the highest level of intensity.

- Joy, with a maximum intensity degree of ecstasy;
- Confidence, with a maximum intensity of admiration;
- Fear, with a maximum intensity of terror;
- Surprise, with a maximum intensity of amazement;
- Sadness, with a maximum degree of intensity of regret;
- Rejection, with a maximum intensity level of abhorrence;
- Anger, with a maximum intensity degree of anger;
- Anticipation, with a maximum intensity of alertness.

To conclude the test, emotional expectations between the trailer and the video game were assessed using an emotional evaluation questionnaire based on obtaining pairs of feelings from Schmidt-Atzert [46]. Data were collected using a 5-point Likert scale where 1 corresponds to the most negative and 5 to the most positive values. The pairs of emotions are as follows: trust–distrust, high quality–low quality, useful–not useful, interesting–boring, familiar–unknown, comfortable–uncomfortable, attractive–unattractive, innovative–conventional, simple–complex, near–far, and fun–not fun.

A quantitative analysis [47] was applied using the initial questionnaire, the emotions questionnaire, the expectations questionnaire, and the data collected with the eyetracker (Tobii Pro Lab (Version v 1.217.49450) [Computer software], Danderyd, Sweden). Tobii Pro Lab allows you to design the test, save the data, and analyze it. Additionally, a qualitative analysis was conducted based on data from the initial questionnaire, the think aloud protocol, and the final interview.

The methodology was designed with the assessment of attention orientation (gaze plot or visual path) and the point of focus in mind, considering that any modification in the characteristics of an object in the graphical interface, such as movement or color, can trigger an involuntary attention orientation reaction that may lead to an emotional response, taking into account other psychological factors, such as motivation or emotional state.

**Figure 7:**
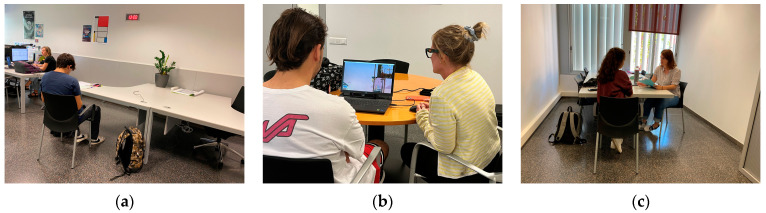
Images of the process carried out at the Polytechnic University of Catalonia (UPC) in Terrassa, Spain. (**a**) The first step is in the discovery phase, (**b**) the second step is in the exploration phase, and (**c**) the third step is in the analysis phase.

Photographs of the process carried out to illustrate the three phases are shown (Figure 7).

As can be interpreted in Figure 7, during the first step, the consultant had an active role during the test explanation and a passive role during the completion of the preliminary questionnaire to allow space for the participant. In step 2, the consultant adopted a facilitator role where they initially explained what is expected from the user and then provided passive support during the video viewing. Finally, in step 3, the consultant assumed the role of an interviewer and actively ensured that the participant felt comfortable responding to all the questions that were posed.

The test was conducted based on viewing the trailer of the indie video game “SpiritFarer”. This video game places the player in the shoes of a girl who must accompany lost souls during their final days before bidding them a final farewell. These souls are represented as anthropomorphic animals and must resolve their unfinished business before moving on to the next life. The game is reminiscent of the Greek myth of Charon, the ferryman of the underworld who carries the souls of the dead to Hades. It is a cozy game [48] designed to address complex themes (death and loss) through gentle aesthetics and gameplay mechanics that do not present hard challenges. The tests were conducted individually, and for each phase, a different classroom was used, with a researcher in each one to streamline the process. Therefore, the sessions were conducted face-to-face, with only the consultant and the participant present.

### 2.4. Participants

This study was conducted with 15 users [49], of which the first 2 were used as pilot users to refine the proposed protocol. The analysis was carried out with 13 potential players, aged between 18 and 25, all of whom were students of the Bachelor’s Degree in Video Game Design and Development at CITM (Center for Image and Multimedia Technology), Polytechnic University of Catalonia (UPC), with knowledge in the audiovisual narrative, game design, and concept art. The time required for each participant was approximately 30 min.

## 3. Findings and Results

Having defined the research objectives, methodology, and techniques, this section presents the results. During the discovery phase, quantitative and qualitative data were collected through the questionnaire. In the exploration phase, we worked with the results collected through the eyetracker device and the think aloud protocol. In the analysis phase, qualitative data were collected through the interview, and quantitative and qualitative data were collected through the emotion assessment questionnaires.

The results are presented organized according to the phase in which they were worked on.

### 3.1. Discovery Phase

#### Pre-Questionnaire

Regarding the preliminary questionnaire, a total of eight questions were asked, focused on gaining a more detailed understanding of video game selection parameters and technical knowledge in the field, taking into account the high homogeneity of the assessed profile.

Concerning the most valued aspects when choosing a video game, eight users selected the trailer, the gaming community’s reviews, and recommendations from friends. Therefore, the significance of trailer content is emphasized.

Regarding the participants’ preferences when selecting a video game, 57.15% showed a preference for AAA games, while 42.85% preferred indie games. In this context, comments were obtained, such as the one from user 8, who stated: “I believe that AAA games are primarily designed for mass consumption and profit, while indies focus more on the experience”. There were also statements like the one from user 10, who mentioned that “the obsession with detail in which AAA games are sometimes made allows them to visually appeal to the player and, therefore, express ideas that are sometimes not possible in an indie game”. User 12 valued “graphics and playing games that many people are playing since I enjoy competitive and multiplayer gaming”.

### 3.2. Exploration Phase

During this phase, the eyetracker device was calibrated for each of the participants. Subsequently, the trailer was displayed.

#### 3.2.1. Eyetracker

**Figure 8:**
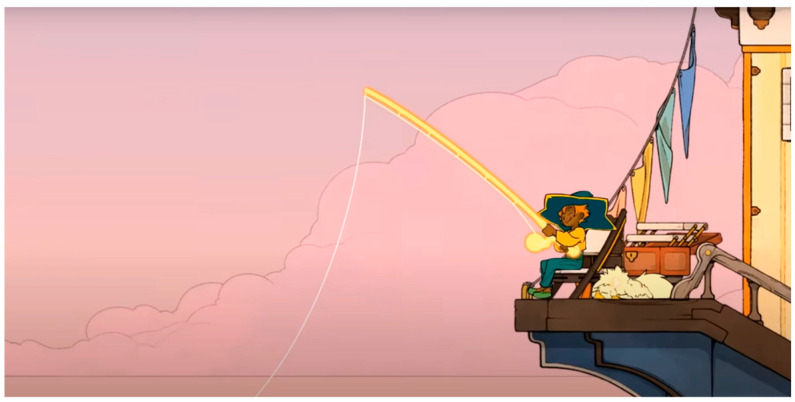
Image representing stimulus 1 introduction.

**Figure 9:**
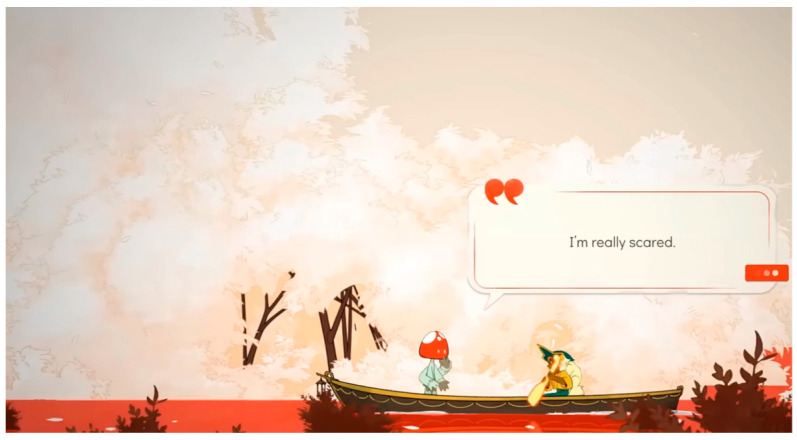
Image representing gameplay stimulus 2.

**Figure 10:**
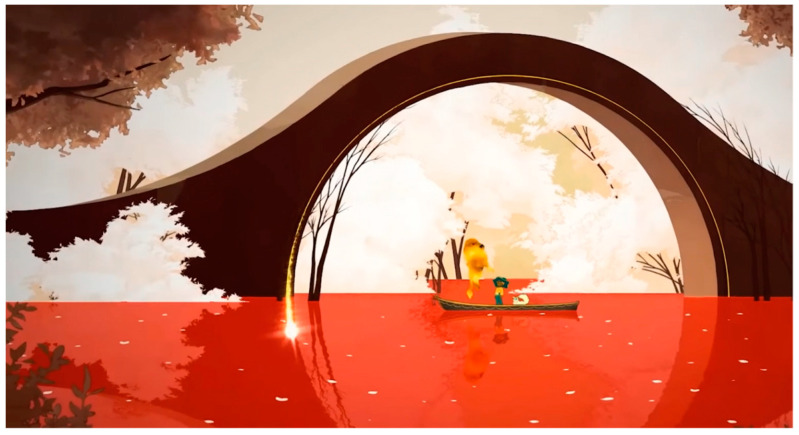
Image representing stimulus 3 of the final part.

The analysis using the eyetracker device focused on three types of stimuli: the introduction, the gameplay, and the end of the trailer, marked as the time of interest (TOI). Various areas of interest (AOI) were indicated for elements visualized in the stimuli that were capable of attracting the participants’ attention, As shown in Figure 8, Figure 9 and Figure 10.

#### 3.2.2. Areas of Interest (AOI)

**Figure 11:**
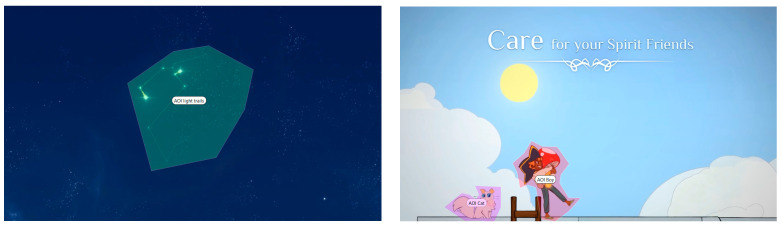
Areas of interest indicated in the middle phase of gameplay.

**Figure 12:**
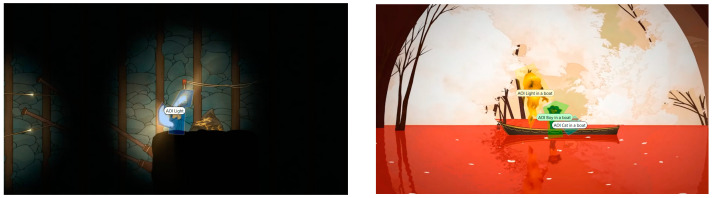
Areas of interest indicated in the final phase.

Areas of interest allow specific information to be collected at specific points in the data visualization. Some of the areas of interest (AOI) marked for this project are shown below, in Figure 11 and Figure 12:

#### 3.2.3. Gaze Plot

The eyetracker visualization shows the sequence and points of fixation in a dynamic environment. The dots’ size indicates the fixation duration, and the number of dots represents the order of these fixations. Gaze plots are used to illustrate the patterns of a single participant.

**Figure 13:**
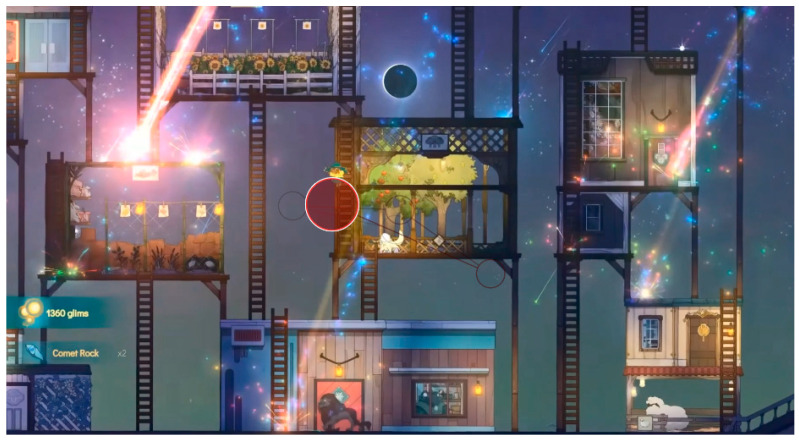
Gaze plot of participant 3 during the visualization of the gameplay part.

As shown in Figure 13, during the gameplay phase, the users remained positioned at the center of the screen and oriented their attention toward the movement zone. The visualization of user 3 is shown as an example.

According to the same criterion, the central zone is the most visualized as it can be interpreted during the whole visualization of the trailer.

**Figure 14:**
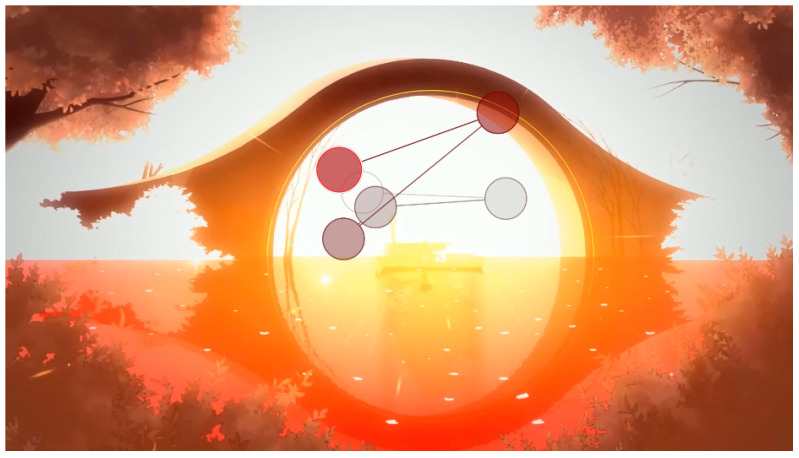
Gaze plot of participant 10 during the viewing of the final stimulus.

Using Figure 14 as an example, the final stimulus of user 10 can be interpreted as the user visualizing the upper zone from the central zone and scrolling the image of the central part.

#### 3.2.4. Heat Map

Heat maps are the sum of the participants’ visualizations. Different colors are used to understand the number and duration of fixations in certain areas. Red indicates a high number of fixations and a long duration; yellow and green vary with decreasing fixations and time. The heat map is based on fixation data consisting of timestamps, duration, spatial location, and X and Y coordinate information. To create a heat map, it is necessary to have the fixation map of the stimulus. For this project, three stimuli were created: introduction, gameplay, and denouement. The numerical value increases according to the fixation points, and the color changes from red to orange to yellow to green. Areas with no heat map indicate non-visible areas, which are also relevant for this study.

**Figure 15:**
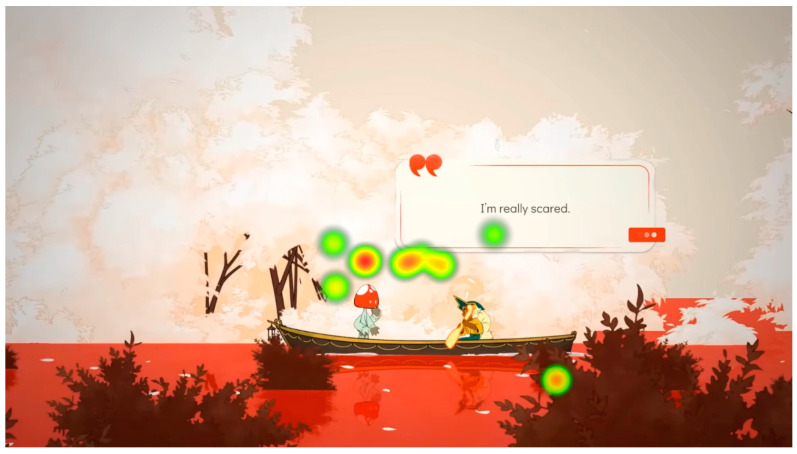
Heat map of all participants during gameplay viewing.

Figure 15 shows the heat map generated among all participants during the gameplay visualization process. The area with the highest degree of visualization is located centrally, while the areas with lower degrees of visualization are situated on the sides. The text, as can be seen, did not become an element of attention, even though it contains an explanatory message.

**Figure 16:**
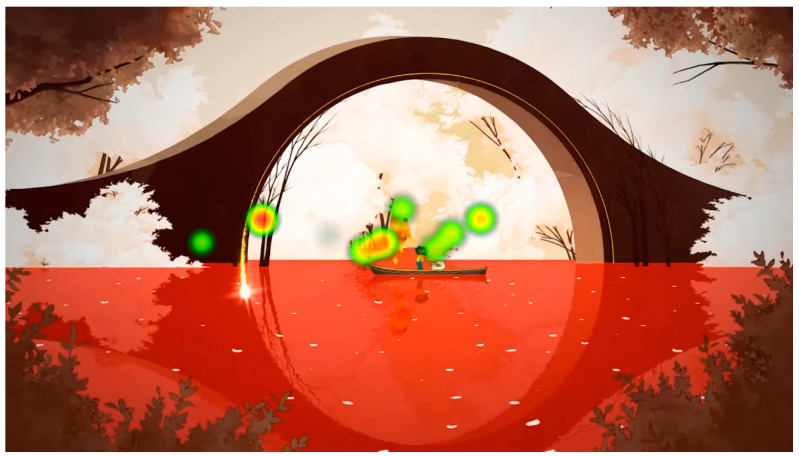
Heat map of all participants during the visualization of the final part.

Figure 16 shows that the visual consistency continues with the visualization of the central area.

#### 3.2.5. Points of Attention

The attention point, according to the attention filter in Tobii Pro Lab, was set with a speed threshold parameter of 100 degrees per second. The number of attention points indicates the order of display, and the size of the circle is related to the duration of the user at that point.

**Figure 17:**
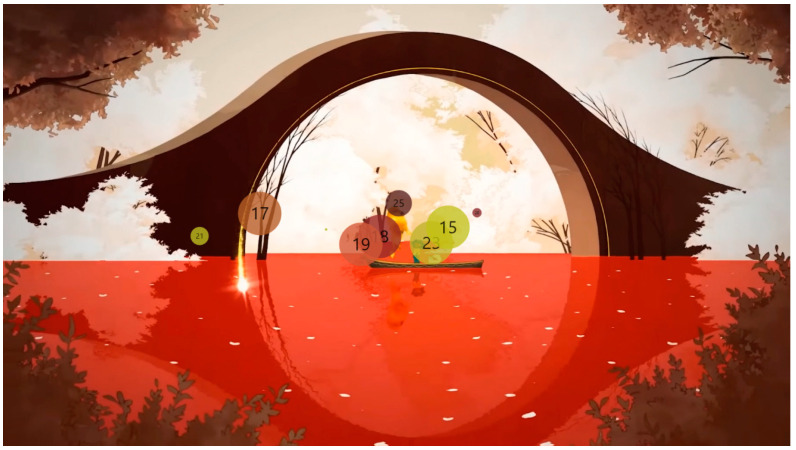
Attention points generated via all participants in the final stimulus.

Figure 17 shows the number of fixations made by all participants, indicating a darker color on the first fixation points and a lighter color on the rest.

#### 3.2.6. Fixing Points

The fixation point, according to the fixation filter in Tobii Pro Lab, was set with a speed threshold parameter at 30 degrees per second, which differed markedly from the 100 degrees per second attention filter. The numbers of the fixation points, like the attention points, indicate the order of display, and the size of the circle is related to the duration of the user at that point.

**Figure 18:**
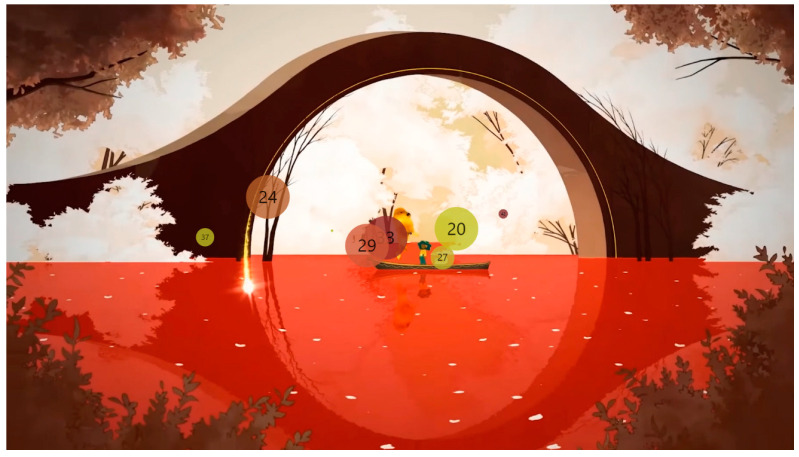
Fixation points generated via all participants in the final stimulus.

Figure 18 shows the number of fixations per point among users, with a continuous result in the central area.

#### 3.2.7. Display Pattern

The visualization pattern was created from the results obtained from all the participants during the visualization at the same point and the order of the visual path they generated.

**Figure 19:**
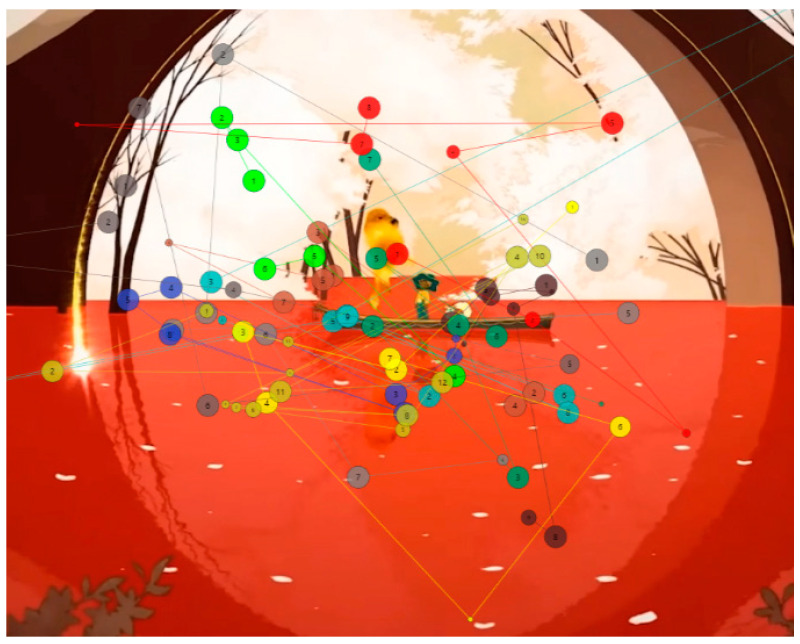
Gaze plot of all participants. Each color represents the visual path of a user.

Figure 19 shows a different color for each participant. The first display point is indicated by a 1, followed by a 2, and then a 3, following the display order.

**Figure 20:**
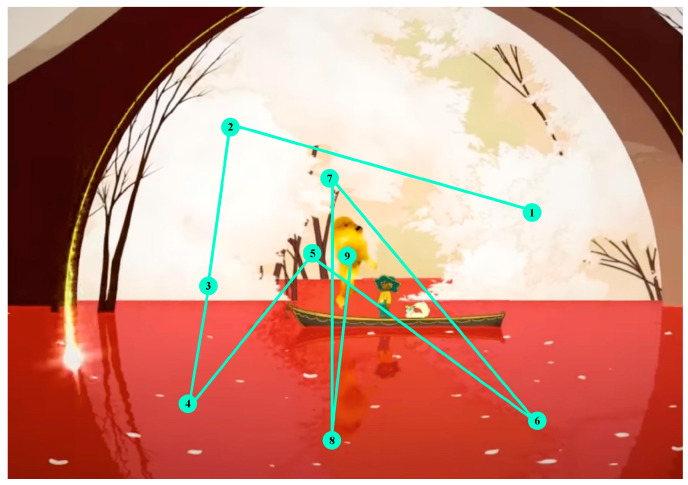
A visualization pattern was generated from the gaze plots of the participants in the previous image.

Figure 20 shows the pattern created by the users, starting in the central part and finalizing in the main element of the interface.

### 3.3. Analysis Phase

Figure 20 shows the pattern created via the users, starting in the central part and ending at the main element of the interface.

#### 3.3.1. Interview

During the interview, the participant was allowed to watch the trailer again so that he/she had no doubts about the information requested, but it was not necessary to remember the video.

The most relevant results are described below:

Question: What is the trailer about?

Interviewer response: “The meaning of the video game trailer is based on learning to say goodbye to loved ones”.

Only one user understood the true meaning of the game; the rest interpreted it as an interesting story. Below are two literals from user 6 and user 11:

- User 6: “The game is about a character who coexists with animals and magical creatures. From what I gathered in the trailer, it seems like she helps them build a boat. However, at a certain point, she aids them in transitioning to the afterlife and accompanies them on a journey to the other side. Ultimately, the animals transform into constellations in the sky”.
- User 11: “It’s about a game that, at first glance, seems to involve gathering different materials to build homes for the animals. However, later in the trailer, it mentions that ‘it’s time to say goodbye’. At that point, it felt like a limbo to me. So, you need to complete a series of missions to fulfil each animal’s final wish before they pass away”.

Question: Looking at the trailer, is it a video game you would play?

Even without fully understanding the true meaning of the narrative, the design and music make users eager to play the video game. Here are some of the evaluations:

- User 4: “Watching the trailer, I would definitely play “Spiritfarer”. Even though the trailer doesn’t tell the story, it does imply there’s an interesting narrative”.
- User 7: “Yes, because I really enjoy these indie game ventures that explore unique, more personal, and intimate themes”.
- User 5: “It’s not the typical game I’d buy, but I wouldn’t say no to it either”.
- User 9: “I don’t enjoy narrative stories, so it’s not a game I would play”.

Question: Does the trailer help you connect with the characters?

In narrative, the actions of the characters are crucial, and several users provide positive evaluations on this aspect.

- User 4: “In a way, it helps me connect, especially with the protagonist”.
- User 12: “It does help, and, above all, one noteworthy aspect beyond aesthetics is the sound in the trailer that aligns perfectly with the different actions you can see in the video. In my case, it greatly aids my connection. I noticed it particularly at the end of the trailer. It complements the narrative exceptionally well and facilitates an emotional connection”.

Negative evaluations:

- User 6: “Not directly with the characters, but yes, with the universe they’ve created. More emotional universes, and the music resonates with me a lot”.
- User 9: “Not for me because I’ve seen more of the interaction between the characters, and there’s clearly a relationship, but it hasn’t helped me connect with them”.

Question: Why do you think the characters are animals?

- User 5: “I think the animal’s character serves to reflect the character’s attitude as if it were a person”.
- User 7: “The characters are animals, and you don’t feel any prejudice as a person. I think it’s a very well-thought-out way to create a very intimate relationship between the player and the NPCs without the player having anything against helping the characters”.

Question: The actual concept of the trailer was explained to the participant—"As you can see, the “Spiritfarer” trailer is about death and knowing how to say goodbye"—and then the participants were asked: What are the elements that conveyed to you that it is about death?

- User 11: “When the lion disappears, they hug, and then you can see a light on the bridge, which represents the circle of life coming to an end. It turns into a constellation”.
- User 6: “For me, the most important thing is the fact that they were taken on a boat. And then they disappear. It reminded me of mythology where there was a ferryman who accompanied people to death. And, in the end, it turns into a constellation”.

#### 3.3.2. Emotions Questionnaire

After the final interview, participants assessed the emotions that arose during the video viewing. To conduct this, the eight emotions of Robert Plutchik, as explained in the previous section, were used.

**Figure 21:**
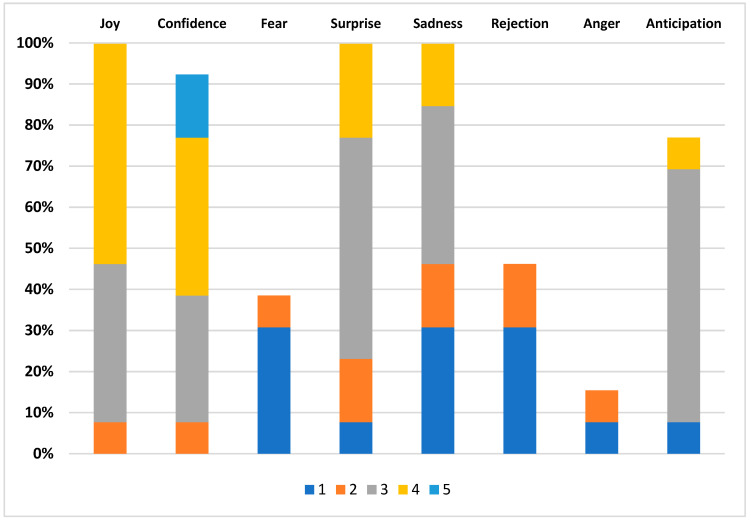
Emotional assessment of the participants regarding the trailer.

Likert scale for assessing each of the emotions indicated in Figure 21:

- Feelings of joy: very little joy (1), little joy (2), joyful (3), very joyful (4), ecstasy (5);
- Feelings of trust: very untrustworthy (1), untrustworthy (2), trustworthy (2), very trustworthy (4), admiration (5);
- Feelings of fear: very little fear (1), little fear (2), fear (3), very much fear (4), terror (5);
- Feelings of surprise: very little surprising (5), little surprising (4), surprising (3), very surprising (4), amazement (5);
- Feelings of Sadness: very little sad (1), little sad (2), Sad (3), very sad (4), very sad (4), very sad (5);
- Feelings of rejection: very little rejection (1), little rejection (2), rejection (3), very much rejection (4), abhorrence (5);
- Feelings of anger: very little anger (1), little anger (2), anger (3), much anger (4), anger (5);
- Feelings of anticipation: very little interest (1), little interest (2), interest (3), much interest (4), alertness (5).

In Figure 21, the analysis of the emotional evaluation of the participants regarding the “Spiritfarer” trailer is presented. In this study, various emotions were quantified, including joy, trust, fear, surprise, sadness, rejection, anger, and anticipation.

The most relevant finding revealed through the data is the prevalence of the emotion of joy, representing 53.85% of the total responses. This finding indicates that the trailer managed to evoke positive emotions in the viewers, such as hope, happiness, and optimism. In the second position, 38.46% of the participants expressed feelings of trust, suggesting that they perceived the trailer as a faithful representation of the game. Surprise is in the fourth position in terms of response frequency, with a total of 53.85%; this indicates that the participants perceived the trailer as something unexpected or atypical. Sadness occupies the fifth position in terms of response frequency, with 38.46% of mentions; this suggests that participants experienced a sense of melancholy or nostalgia when watching the trailer, especially in its final segment.

At the opposite end of the spectrum, rejection is positioned in the sixth place with a single mention, suggesting that only one participant found the trailer unpleasant or unacceptable. On the other hand, anger ranks seventh and last in response frequency, with only two mentions, indicating that two participants found the trailer frustrating or annoying.

Overall, the results of the graph provide a positive perspective. They suggest that the “Spiritfarer” trailer managed to generate a positive emotional response in the audience, a significant achievement considering that the game addresses themes that are often delicate and challenging.

The trailer is capable of evoking a wide range of emotions, spanning from joy and trust to fear and sadness. This result suggests that the trailer can successfully connect with a diverse audience. Additionally, for the majority of viewers, the trailer managed to generate a positive emotional response; this indicates that the trailer was effective in communicating the message and tone of the game, a crucial aspect in promoting works that deal with sensitive themes.

#### 3.3.3. Narrative Questionnaire

The narrative questionnaire collected the emotions that arose during the three proposed stimuli: introduction, gameplay, and the final part. The introduction was configured between times 00:00 and 00:26, the gameplay between 00:27 and 01:50, and the final part between 01:51 and 02:42.

Table 1 shows the results of the participants’ assessments based on the different parts of the narrative.

The emotions are consistent with the assessments from the other questionnaires and comments in all phases of viewing. Positive emotions were primarily shown at the beginning, and towards the end, when the participants began to understand the content, they transitioned to feelings of sadness.

**Figure 22:**
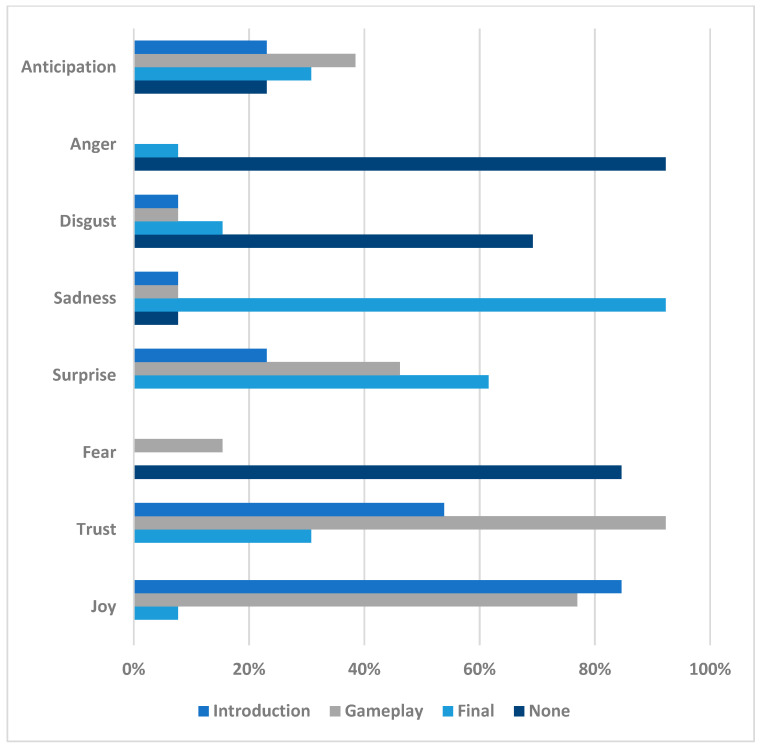
Visualization of the collected emotional data.

In Figure 22, the results are presented and analyzed visually, allowing us to gain a deeper understanding of the key findings and trends extracted from the data.

- Introduction: In the introduction phase of the trailer, the predominant emotion was joy, reaching 84.62%. This response was triggered through the presentation of images and music that created an expectation in the viewer, making them look forward with interest to what would happen next. Additionally, in this stage, other emotions, such as trust (53.85%), were aroused through the display of positive images like natural landscapes and animals. Anticipation and surprise (23.08%) were also present, stemming from the perception that the trailer was well-crafted and augured a high-quality production. Lastly, sadness and disgust reached 7.69%. Fear and anger (0.00%) were not present in this phase, as they are emotions generally associated with negative or threatening situations.
- Gameplay: In the gameplay section, the most dominant emotion was trust (92.31%) because the gameplay presented exciting and fun images and actions. Other emotions that manifested in this phase were joy (76.92%) and surprise (46.15%), resulting from the feeling that the game would be entertaining and challenging. Anticipation (38.46%) was present due to the expectation of what would happen next. Fear (15.38%), sadness, and disgust (7.69%) were experienced in some action scenes but did not prevail as the predominant emotion. Finally, anger was the emotion experienced the least, representing 0%.
- Final: In the conclusion of the trailer, the most prominent emotion was sadness (92.31%); this was due to the unexpected and disappointing outcome for some viewers. Another emotion present in this phase was surprise (61.54%), generated through the feeling of loss or disappointment. Trust (30.77%) was present, stemming from dissatisfaction with the conclusion. Disgust represented 15.38%, while anger and joy were both at 7.69%. Fear (0.00%) did not manifest at this stage, as the story concluded.
- The analysis of the emotion questionnaire presented in the image reveals that the trailer elicited a range of positive emotions, such as joy, confidence, and anticipation. However, the conclusion of the trailer generated a negative emotion, anger, in some viewers.

#### 3.3.4. Expectation Questionnaire

The expectations questionnaire helps to understand the importance of the trailer’s narrative in relation to the expected gameplay of the video game. Table 2 shows the average percentage of each of the emotions, with 100% corresponding to a positive evaluation and 0% to a negative evaluation.

The results, based on pairs of emotions, highlight positive evaluations ranging from comfortable to innovative, showing good data regarding expectations for the video game.

**Figure 23:**
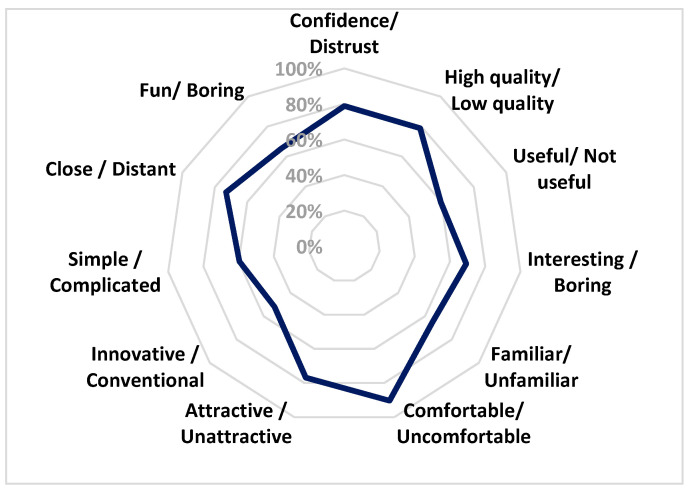
Plot of all pairs of feelings.

In Figure 23, critical information regarding participants’ perceptions of a specific product or experience is revealed and interpreted. The questionnaire addressed a range of emotional and practical dimensions that shed light on how users perceived the product and, consequently, influenced its potential reception.

First and foremost, confidence and high quality of the product stand out, both at a robust 78.85%. This finding indicates the trust participants had in the product and the perception of it being of high quality, essential factors for its acceptance and satisfaction. The perception of utility, while positive at 59.62%, suggests that approximately half of the participants considered the product useful; this underscores the need to enhance users’ perceived utility, which could increase adoption and satisfaction. The factor of interest is also crucial, with the majority of participants rating the product as interesting, at 69.23%, which is a positive indicator. It implies that the product has the ability to maintain users’ attention, a key element for its success. The familiarity with the product is noteworthy, with 65.38% of participants perceiving it as familiar. This familiarity can be a significant asset, as known elements tend to generate a more positive reception. The perception of comfort is exceptionally high, reaching 90.38%; this is fundamental for an effective user experience, as comfortable users are more likely to use and recommend the product. The visual attractiveness of the product is supported by 76.92% of participants rating it as visually attractive; this is a positive sign in terms of the product’s aesthetics. The perception of innovation, at 51.92%, is in the middle, suggesting that some participants viewed the product as innovative while others considered it more conventional; this underscores the diversity of opinions regarding the product’s originality. The perception of simplicity and complexity is balanced at around 59.62%, highlighting the importance of finding a balance in product design to cater to various audience preferences. The feeling of proximity, at 73.08%, indicates that most participants felt close to the product, suggesting a sense of connection or involvement.

Finally, the perception of enjoyment exceeds that of boredom at 65.38%, which is essential for retaining and engaging users.

#### 3.3.5. Relationships between Points of Attention, Emotions, and Storytelling Structure of the Trailer

Based on the above results, Table 3 shows a comparison between the predominant emotions regarding the storytelling of the trailer, the most relevant comments that emerged in the final interview, and the comments that emerged during the viewing of the trailer. In order to finish linking the information collected, a comparison was made between the points of attention of the three stages of storytelling and the comments made by the related participants.

In relation to the data presented in the previous sections of the analysis, it is possible to establish some connections between the narrative structure of the trailer, the predominant emotion experienced by the participants in each part of the trailer (introduction, gameplay, and ending), and the statements they made both in the final interviews and during the think aloud process. The information provided by the participants in the pre-questionnaire also helps to clarify the interpretation of the data provided in the next paragraphs.

As previously stated, the predominant emotion was joy in the first part of the trailer, consisting of the introduction. The trailer introduces the main character of the game (Stella), the ship she will inhabit during the adventure, and some of the non-player characters with whom the player will have to interact with.

Based on the final interview participants’ contributions (3.3.1), it is clear that the elements that most caught their attention were the game characters and the aesthetics. The initial questionnaire reinforces this idea, as participants mostly indicated that the aspects they considered most important in the video game trailer were the visual and sound aesthetics (100% of the responses), followed by the characters and their design (85.7% of the responses). Statements collected in the think aloud process also point to this idea: “It looks interesting at first sight. The art style looks cool” (U4); “I feel joy, excitement” (U14).

Taking into account the predominant emotion in the introduction of the trailer, the data collected in the initial and final interview, and the think aloud process, the emotion of joy can be mainly attributed to the visual and sound aesthetics and the character design.

Regarding the second part of the trailer, which shows the gameplay of the game, the predominant emotion was confidence. This part of the trailer shows the main actions to be carried out by the player to achieve the goals set by the game.

The comments provided in the final interview (3.3.1) confirm that the participants understood the most frequent game mechanics and what the player can do in the game. In addition, during the think aloud process, a large portion of the users identified some features of the game and expressed that the mechanics reminded them of games they had already played: “It doesn’t feel like there are any enemies” (U6); “There are times when it reminded me of when I used to play. A platformer, satisfying just because it allowed me to jump. It was something I hadn’t felt in any game” (U7); “It reminds me a lot of the game GRIS (U3)”.

Therefore, from this information, the emotion of confidence in the gameplay part of the trailer can be mainly associated with the fact that the participants found game mechanics that were familiar to them because they were similar to the ones in other games they already knew.

Finally, and in relation to the last part of the trailer, namely the ending, the predominant emotion was sadness. This part of the trailer contains revealing texts of the main narrative premise of the game (for example: “learn how to say goodbye”) and excerpts from the game in which the main character says goodbye to the souls that will pass to the afterlife. One of the participants, in fact, recognized in these excerpts the reference to the myth of Charon.

The final interviews (3.3.1), as well as the comments provided during the think aloud process, allow us to confirm that the participants perceived that the game’s story becomes sad: “Now it looks sad. Very cool animations. It’s like they take the animals away when they die” (U6); “When you have to say goodbye it’s really hard” (U7).

The emotion of sadness, therefore, can be associated with the narrative premise and the idea of loss and death.

**Figure 24:**
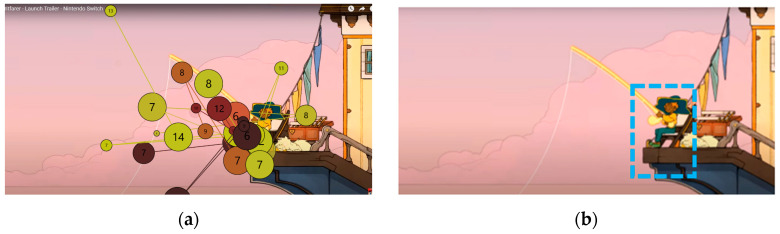
The attention points of all participants in the introductory stimulus. (**a**) The attention points of all participants, each of them having a different color; (**b**) the area where most of the attention points are focused.

**Figure 25:**
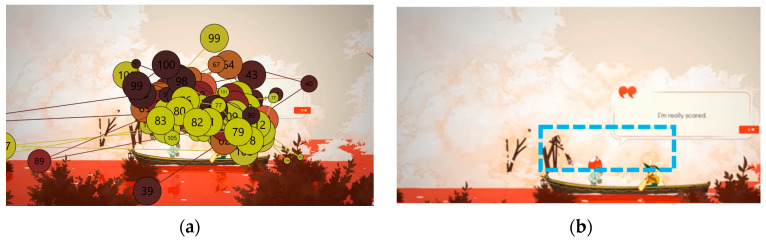
The attention points of all participants in the gameplay stimulus. (**a**) The attention points of all participants, each of them having a different color; (**b**) the area where most of the attention points are focused.

**Figure 26:**
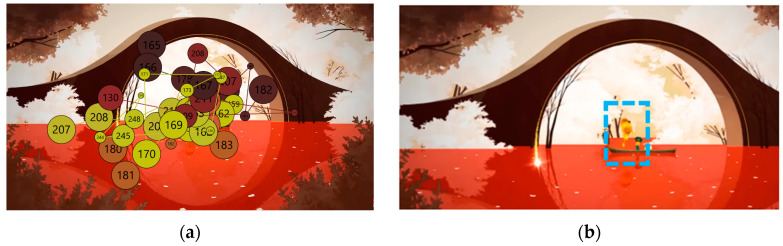
The attention points of all participants in the final stimulus. (**a**) The attention points of all participants, each of them having a different color; (**b**) the area where most of the attention points are focused.

The predominant visual focus is linked to the narrative elements as they frame the points of attention. The trailer guides users’ viewing to emphasize key points you want to convey and emotions you want to be understood. Figure 24, Figure 25 and Figure 26 below visually indicate the points of focus at specific moments in the narrative.

During the viewing of the introductory part, with an example shown in Figure 24, the main character is visually followed, and the narrative is given importance; users reported comments such as: “Very fluid viewing time”, “Looks interesting right off the bat”, “The art style looks cool”, “At first I get a sense of knowing what is going on, I’m very attracted to the way the world is presented and the material you can build”, “It looks very nice, the colour appeals to me quite a lot. The aesthetic seems minimalistic, as I like it. I think the game is very much about what it’s trying to express, and the lighting gives it a touch that stands out”. The sound and aesthetics are also relevant to reinforce the message: “I like the sound and the movement”, “I like the music already, I haven’t seen the trailer”, “I like the aesthetics”. The emotion they convey the most is that of joy: “I feel joy, excitement”.

During the visualization of the gameplay part, with an example shown in Figure 25, participants did not understand the real meaning of the game and interpreted few mechanics: “Mechanics are not simple. Enriching elements that make you think about many things”, “The game seems complete”, “There don’t seem to be enemies”, “The game is for enjoyment. Any task needs an evolution. It seems to have the game very controlled. There are times when it reminded me of when I used to play the game. A platformer, just the satisfaction of jumping was something I hadn’t felt in any game”. “At the moment it doesn’t let me understand what the game is about. There are a lot of distracting elements and I don’t really understand the purpose of the game, what I have to achieve, I understand that I have to build my boat. I don’t understand the objective, is the ship a metaphor, is it dying and I have to save it?”, “It seems to be about getting things”, “It seems to be about doing tasks”, “From what I understand, it’s a game about spirits, but I guess each spirit will have its own mission”. Although in this case, the aesthetics and animation do not seem to help resolve doubts: “I like it, different things are shown, maybe too many things, I don’t understand the story”, “I am struck by the things that are shining, but I don’t know what’s going on”, “The lighting is very much about what you want to see. At the moment it is a soft aesthetic, the background is quite separate from the objects that move in front of everything and the attention is drawn to the objects”.

During the viewing of the denouement part, with an example shown in Figure 26, the participants began to understand that something is ending, and the most conveyed emotion was sadness: “It wouldn’t be the game I would play every week, but on a weekend, being quiet and enjoying it, I’ve been wanting to know more”, “It looks like a game that I don’t know if it makes you cry but at least it makes you feel things, it has a powerful story”, “Now it looks sad, very cool animations, it’s like they take the animals away when they die. You have cool shots and the pace of the trailer is very appropriate”, “When you have to say goodbye it’s very hard. It’s always delayed so you don’t have to say goodbye”, “It’s very nice, but it doesn’t give me the feeling they want to give. I understand that you go to heaven and you collect animals?”, “It looks like someone dies”, “It looks like we’re moving on to something sadder”.

## 4. Discussion

In this study, emotions and visual attention are analyzed using different techniques from a dual perspective. On the one hand, the results obtained from the interview and the emotional assessment questionnaires allow an adjustment towards the users’ perceptions, and the assessment using the eyetracker device allows us to obtain an objective and real metric of visual attention.

During the gameplay phase, as an essential part of the trailer’s narrative, several explanatory sentences are included, but participants do not visualize these as they focus on the images. In the initial part of the narrative, the most perceived emotion is joy, contrasting with the trust emotion generated during gameplay viewing and the final emotion of sadness towards the end, once participants have understood the game’s narrative. Visual attention is entirely centered on the middle part of the screen, highlighting areas not visible in the rest of the interface. There are no differences between fixation points and points of attention, likely because the points of attention are detailed through obtaining more screen time; this could be due to the multitude of elements appearing on the screen and the clear placement of primary elements in the central part of the screen.

Regarding the visual orientation of all participants, a single pattern based on the visual journey has been created due to the consistency in viewing. The eyetracker shows data from the gaze plot and heatmap in the central area during the gameplay phase, which is linked to the feeling of sadness, indicative of the type of video game content.

As suggested in the initial survey of the test, only one participant understood the trailer’s metaphor, so emotions begin with enjoyment and end with sadness, with surprise being prominent in the final part when the narrative becomes evident. Regarding expectations of the video game, a 90.38% comfortable rating is observed, indicating a perception of a pleasant, tranquil game without challenging hurdles; this is followed by 78.85% confidence and high quality, interesting, attractive, and less innovative, which highlights the gameplay narrative, indicating a context of familiar mechanics and platforms. During the video, there are text fragments with explanations that are not being read but may be relevant to understanding the content. Therefore, the narrative is linked only to images, not text. There is no difference between fixation points and points of attention as the orientation is centered in the design. In this trailer, image composition has been a significant factor.

The results related to visual attention conform to Thomsen and Heiselnerg’s two-peak structure, where the first peak is based on the level of excitement in the initial part of the trailer, and the second peak occurs at the conclusion. According to the findings, factors such as motion, visual content hierarchy, and color contrast serve as external stimuli that capture involuntary attention. The design structure, focusing on specific characters and minimizing visual clutter, promotes increased user concentration. The game elements’ design enables all participants to consistently follow the basic attention process: detecting the interface, selecting elements of interest, directing attention along a uniform visual path, and remaining vigilant over the same area of interest.

This research represents an innovative and non-intrusive approach to the analysis of perceived storytelling and the assessment of visual attention linked with emotions in the context of video game trailers. It contributes to advancing the study of video game trailers, as studies employing mixed methodologies for evaluation predominantly rely on obtaining results from physiological skin response measures associated with emotional parameters.

## 5. Conclusions

This study is presented as a pilot study where experts in video games can utilize an easily applicable methodology without delving into specific knowledge across various domains. Presently, efforts continue to advance marketing within the realm of video games, particularly in enhancing the value of trailers as a fundamental tool for attracting future players.

This research has allowed us to innovate the assessment of emotions through the design of a non-intrusive mixed methodology: assessment of emotions using Robert Plutchik’s wheel of emotions through the eight emotions: joy, confidence, fear, surprise, sadness, rejection, anger, and anticipation, and the link of these emotions with the storytelling that is visualized before the introduction, the gameplay, and the ending. The data collected from user responses are related to the objective information collected using the eyetracker device: visual attention, fixation, gaze plot, visualization pattern, heatmap, and blind zones, designing the stimuli using the same storytelling: introduction, gameplay, and ending.

In this research, the Schmidt-Atzert emotional appraisal questionnaire is also applied in a novel way, using the pairs of positive and negative emotions: confidence–distrust, high quality/low quality, useful/not useful, interesting/boring, familiar/unfamiliar, comfortable/uncomfortable, attractive/unattractive, innovative/conventional, simple/complicated, close/distant, fun/boring. This is applied for the assessment of the game expectation and not for the assessment of the user experience as it is normally used, allowing the obtainment of very relevant and consistent data regarding the perceived experience in the visualization of an informative trailer before the expectation of the video game.

Ongoing work aims to refine the method to provide more detailed information that may be relevant to the results; this includes distinguishing between types of trailers, whether they include gameplay footage or not, and delving further into the influence of audio and aesthetics on visual attention assessments.

## Figures and Tables

**Table 1:** Results of the assessments of all participants regarding the emotions at different points in the trailer’s narrative.

Section	Joy	Trust	Fear	Surprise	Sadness	Disgust	Anger	Anticipation
Introduction	84.62%	53.85%	0.00%	23.08%	7.69%	7.69%	0.00%	23.08%
Gameplay	76.92%	92.31%	15.38%	46.15%	7.69%	7.69%	0.00%	38.46%
Final	7.69%	30.77%	0.00%	61.54%	92.31%	15.38%	7.69%	30.77%
None	0.00%	0.00%	84.62%	0.00%	7.69%	69.23%	92.31%	23.08%

**Table 2:** Results of the emotion ratings in the expectations questionnaire.

	Average
Confidence/Distrust	78.85%
High quality/Low quality	78.85%
Useful/Not useful	59.62%
Interesting/Boring	69.23%
Familiar/Unfamiliar	65.38%
Comfortable/Uncomfortable	90.38%
Attractive/Unattractive	76.92%
Innovative/Conventional	51.92%
Simple/Complicated	59.62%
Close/Distant	73.08%
Fun/Boring	65.38%

**Table 3:** Comparative table between predominant emotions when viewing the trailer, final interview, and thinking aloud during the display.

Introduction	Gameplay	Final
Predominant emotions when viewing the trailer		
Joy	Trust	Sadness
Final interview		
What is the trailer about?		
“The game is about a character who coexists with animals and magical cre + B14atures” (U6).	“It seems like she helps them build a boat” (U6).	“She aids them in transitioning to the afterlife and accompanies them on a journey to the other side. Ultimately, the animals transform into constellations in the sky” (U6).
	“It’s about a game that, at first glance, seems to involve gathering different materials to build homes for the animals” (U11).	“Later in the trailer, it mentions that ‘it’s time to say goodbye’. At that point, it felt like a limbo to me. So, you need to complete a series of missions to fulfil each animal’s final wish before they pass away”(U11).
Looking at the trailer, is it a video game you would play?		
		“I really enjoy these indie game ventures that explore unique, more personal, and intimate themes” (U7).
Does the trailer help you connect with the characters?		
		“It does help, and, above all, one noteworthy aspect beyond aesthetics is the sound in the trailer that aligns perfectly with the different actions you can see in the video. In my case, it greatly aids my connection. I noticed it particularly at the end of the trailer. It complements the narrative exceptionally well and facilitates an emotional connection”(U12).
What are the elements that conveyed to you that it is about death?		
		“When the lion disappears, they hug, and then you can see a light on the bridge, which represents the circle of life coming to an end. It turns into a constellation” (U11).
		“For me, the most important thing is the fact that they were taken on a boat. And then they disappear. It reminded me of mythology where there was a ferryman who accompanied people to death. And, in the end, it turns into a constellation” (U6).
Thinking aloud during the display		
“At first glance it looks interesting. The art style looks cool” (U4).	“The animation is very well done. There don’t seem to be any enemies” (U6).	“It looks like a game that I don’t know if it makes you cry but at least it makes you feel things, it has a powerful story” (U4).
“I feel joy, emotion” (U14).	“The game is for enjoyment. Any task needs an evolution. You seem to have the game very much under control. There are times when it reminded me of when I was playing. A platformer, just the satisfaction of jumping was something I hadn’t felt in any game” (U7).	“Now it looks sad. Very cool animations. It’s like they take the animals away when they die. You have cool shots and the pacing of the trailer is very appropriate” (U6).
	“The mechanics are not simple. Enriching elements that make you think about many things. It reminds me a lot of Gris game” (U3)	“When you have to say goodbye it is very hard. Everything is always delayed so that you don’t have to say goodbye” (U7).
	“It seems to be about doing homework” (U14).	“We seem to be moving on to something sadder” (U14).
